# How Aconiti Radix Cocta can Treat Gouty Arthritis Based on Systematic Pharmacology and UPLC-QTOF-MS/MS

**DOI:** 10.3389/fphar.2021.618844

**Published:** 2021-04-30

**Authors:** Xietao Ye, Jianxiong Wu, Dayong Zhang, Zelun Lan, Songhong Yang, Jing Zhu, Ming Yang, Qianfeng Gong, Lingyun Zhong

**Affiliations:** ^1^ Pharmacy College, Jiangxi University of Traditional Chinese Medicine, Nanchang, China; ^2^ Sichuan New Lotus Chinese Herbal Medicine, Chengdu, China

**Keywords:** aconiti radix cocta, gouty arthritis, systems pharmacology, active ingredients, mechanism of action

## Abstract

**Background:** Gouty arthritis (GA) is a common metabolic disease caused by a long-term disorder of purine metabolism and increased serum levels of uric acid. The processed product of dried root of *Aconitum carmichaeli* Debeaux (*Aconiti Radix* cocta, ARC) is used often in traditional Chinese medicine (TCM) to treat GA, but its specific active components and mechanism of action are not clear.

**Methods:** First, we used ultra-performance liquid chromatography-quadrupole/time-of-flight tandem mass spectrometry to identify the chemical spectrum of ARC. Based on this result, we explored the active components of ARC in GA treatment and their potential targets and pathways. Simultaneously, we used computer simulations, *in vitro* cell experiments and animal experiments to verify the prediction results of systems pharmacology. *In vitro*, we used aurantiamide acetate (AA) to treat monosodium urate (MSU)-stimulated THP-1 cells and demonstrated the reliability of the prediction by western blotting and real-time reverse transcription-quantitative polymerase chain reaction (RT-qPCR). ELISAs kit were used to measure changes in levels of proinflammatory factors in rats with GA induced by MSU to demonstrate the efficacy of ARC in GA treatment.

**Results:** Forty-three chemical constituents in ARC were identified. ARC could regulate 65 targets through 29 active components, and then treat GA, which involved 1427 Gene Ontology (GO) terms and 146 signaling pathways. Signaling pathways such as proteoglycans in cancer, C-type lectin receptor signaling pathway, and TNF signaling pathway may have an important role in GA treatment with ARC. *In silico* results showed that the active components songoramine and ignavine had high binding to mitogen-activated protein kinase p38 alpha (MAPK14) and matrix metallopeptidase (MMP)9, indicating that ARC treatment of GA was through multiple components and multiple targets. *In vitro* experiments showed that AA in ARC could effectively reduce expression of MAPK14, MMP9, and cyclooxygenase2 (PTGS2) in THP-1 cells stimulated by MSU, whereas it could significantly inhibit the mRNA expression of Caspase-1, spleen tyrosine kinase (SYK), and PTGS2. Animal experiments showed that a ARC aqueous extract could significantly reduce expression of tumor necrosis factor (TNF)-α, interleukin (IL)-1β, and intereleukin (IL)-18 in the serum of GA rats stimulated by MSU. Hence, ARC may inhibit inflammation by regulating the proteoglycans in cancer-associated signaling pathways.

**Conclusion:** ARC treatment of GA may have the following mechanisms, ARC can reduce MSU crystal-induced joint swelling, reduce synovial tissue damage, and reduce the expression of inflammatory factors in serum. AA in ARC may inhibit inflammation by regulating the protein expression of MAPK14, MMP9, and PTGS2 and the mRNA expression of caspase-1, SYK, and PTGS2.

## Introduction

Gouty arthritis (GA) is a rheumatic disease that often causes lesions and inflammatory reactions due to deposition of urate crystals in tissues (e.g., joint capsule, bursa, cartilage) (Lu et al., 2013; Ottaviani et al., 2013). In acute attacks of GA, non-steroidal anti-inflammatory drugs and colchicine are given, but side effects (rash, edema, diarrhea, myocardial infarction) can occur. In addition, GA is often accompanied by renal, cardiovascular, or gastrointestinal diseases, which also limit clinical application of these drugs (Finkelstein et al., 2010; Zavodovsky and Sivordova, 2018). Accordingly, a new therapeutic candidate with more efficacy and safety is indeed in demand for GA treatment.

Traditional Chinese medicine (TCM) has been practiced in East Asia for centuries (Yue et al., 2019). Increasingly Chinese herbal medicines (or their extracts) are being included in treatment regimens in Western countries (Kuo and Chou et al., 1995; Kong et al., 2004). In TCM theory, formulations must be processed and their safety demonstrated before they can be used clinically (Chen et al., 2020; Committee, 2020).

The processed product of dried root of *Aconitum carmichaeli* Debeaux (*Aconiti Radix* cocta, ARC) can be used to eliminate wind, remove dampness, disperse cold, relieve pain, and warm meridians. Originally in the Eastern Han Dynasty of China (24-220 AD), ARC was recorded by “Shennong Materia Medica” (Shengnong Ben Cao Jing), the earliest Pharmacopeia of China, and was described for its medicinal effect against chronic arthritic diseases by “Treatise on Febrile Diseases” (Shang Han Lun), a classical TCM book (Tong et al., 2013). Its anti-rheumatism and anti-arthralgia effects exerted through dispelling the wind-cold-damp evil have been presented in the Chinese Pharmacopeia (Committee, 2020). Modern studies have evidenced that such effects of ARC in combination with other herbs have anti-GA property in the therapy of patients (Ameri, 1998), indicating its promising therapeutic potential against GA, however the specific mechanism of action remains unclear.

ARC is highly toxic before processing and should not be eaten (Yue et al., 2007). Patients with root of *Aconitum carmichaeli* Debeaux poisoning typically present with a combination of neurological, cardiovascular, gastrointestinal and other signs and symptoms (e.g., paresthesia and numbness of face, palpitations, sinus tachycardia) (Chan, 2009). The raw root of *Aconitum carmichaeli* Debeaux, including aconitine, mesaconitine, and hypaconitine, which have median lethal dose (LD_50_) values of 1.8, 1.9, and 5.8 mg/kg, respectively, for oral administration in mice. Through this heat-processing, aconitine, mesaconitine, and hypaconitine are hydrolyzed at the carbon 8-position to become benzoylaconine, benzoylmesaconine, and benzoylhypaconine, respectively. It is reported that the LD_50_ values of benzoylaconine, benzoylmesaconine, and benzoylhypaconine are 1.5, 0.81, and 0.83 g/kg, respectively (Tanimura et al., 2019). The toxicity of ARC is reduced and its efficacy increased after processing (Zhang et al., 2017).

Ultra-performance liquid chromatography-quadrupole/time-of-flight tandem mass spectrometry (UPLC-QTOF-MS/MS) has the advantages of fast separation, high sensitivity, and can provide accurate relative molecular mass. UPLC-QTOF-MS/MS is used commonly for analyses of the chemical constituents of complex TCM formulations (Wang et al., 2020). Therefore, to better elucidate the main pharmacophoric components in ARC, we utilized UPLC-QTOF-MS/MS for identification.

**FIGURE 1:**
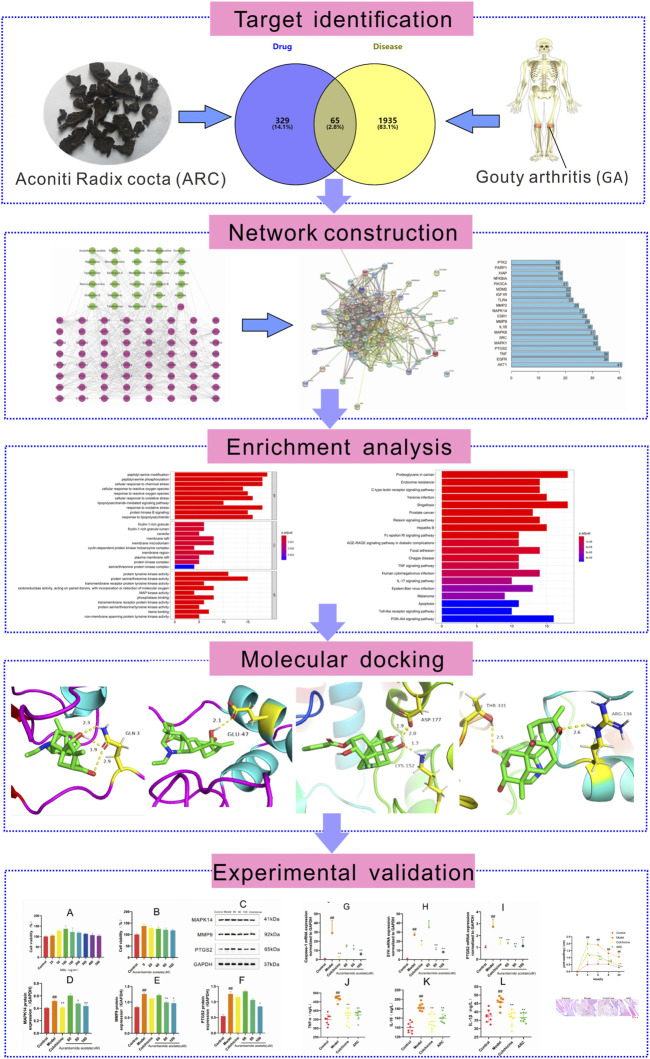
Systems pharmacology-based strategy to study the molecular mechanism of ARC in treatment of gouty arthritis.

In recent years, systems pharmacology has been used widely in TCM research. This approach enables systematic determination of the effects and mechanisms of drugs used to treat complex diseases at molecular, cellular, tissue, and biological levels (Yang et al., 2019). Several studies have demonstrated the exact efficacy of ARC for GA, but its specific mechanism of action is not clear. We used systems pharmacology to analyze the active ingredients, drug targets, and key pathways in ARC treatment **(** Figure 1 **)**.

## Materials and Methods

### Ethical Approval of the Study Protocol

The animal study was reviewed and approved by the Research Ethics Committee of the Jiangxi University of Traditional Chinese Medicine (approval number: JZLLSC20200009). NO potentially identifiable human images or data is presented in this study.

### Chemicals, Agents and Materials

Dried root of *Aconitum carmichaeli* Debeaux (chuan-wu in Chinese, CW, batch NO. 20181011), *Zingiber officinale* Roscoe (sheng-jiang in Chinese, SJ, batch NO. 20190219), dried root of *Glycyrrhiza glabra* L. (gan-cao in Chinese, GC, batch NO. 20181224) and *Saponaria officinalis* L. (zao-jia in Chinese, ZJ, batch NO. 20190101) were purchased from Xujiang Ecological Technology (Nanchang, China)*.* UHPLC-MS-grade methanol and formic acid were purchased from Anaqua Chemicals Supply (ACS, Boston, MA, United States), UHPLC-MS-grade acetonitrile was obtained from Merck Drugs and Biotechnology (Darmstadt, Germany). Ultrapure water was provided by Watson's Food and Beverage (Guangzhou, China).

As reference standards, four pure compounds were used (purity≥98%). Among these compounds, benzoylmesaconine (25), benzoylaconine (26), Benzoylhypacoitine (28) were purchased from Chendu Chroma Biotechnology Co., Ltd. (Sichuan, China). Aurantiamide acetate (41) was purchased from Must Biotechnology (Chengdu, China).

THP-1 cells were purchased from Beina Chuanglian Biotechnology (Beijing, China). A Bicinchoninic Acid (BCA) protein quantification kit, Dulbecco’s modified Eagle’s medium and Cell Counting Kit (CCK)-8 kit were obtained from Beijing Solaibao Technology (Beijing, China). Phorbol-12-myristate-13-acetate (PMA) was purchased from Abcam (Cambridge, United Kingdom). Fetal bovine serum was from Zhejiang Tianhang Biotechnology (Huzhou, China). Monosodium urate (MSU) and colchicine were purchased from Sigma–Aldrich (Saint Louis, MO, United States). Anti-glyceraldehyde 3-phosphate dehydrogenase (GAPDH) antibody and anti-matrix metallopeptidase (MMP)9 antibody were obtained from Abcam. Rabbit anti-cyclooxygenase-2 antibody was purchased from Beijing Boosen Biotechnology (Beijing, China). An ECL chemiluminescence kit was from Millipore (Burlington, MA, United States). A tumor necrosis factor (TNF)-α kit, interleukin (IL)-1β kit, and IL-18 kit were purchased from Jiangxi Boyinda Biotechnology Co., Ltd. (Nanchang, China).

### Preparation of ARC Extracts

CW was soaked in water (which was changed twice a day) for 2 days until its core was completely moist. CW was removed, and SJ, GC, and ZJ added. The water was changed and CW boiled for 4 h until its core was cooked completely. It was boiled for 2 h, removed, dried to 60% and cut into thin slices, followed by further drying (Administration, 2009). Per 100 kg of CW, we used 2 kg of SJ, 5 kg of GC, and 1 kg of ZJ.

The dried sample was pulverized into powder and passed through a size-3 sieve. We weighed 1 g of powder accurately, added 30 ml of methanol, and undertook ultrasonic extraction for 20 min. After cooling, the weight loss was compensated, followed by filtering. The filtrate was diluted 3-fold, and passed through 0.22-μm microporous filter membrane (Shan et al., 2012).

### UPLC-QTOF-MS/MS

A Triple TOF™ 5600 liquid chromatography high-resolution tandem mass spectrometer (AB SCIEX, Framingham, MA, United States) equipped with a DuoSpray™ ion source, 30-A liquid chromatography system, and Acquity UPLC BEH C18 column (2.1 mm × 100 mm, 1.7 μm; Waters, Milford, MA, United States) was used. The mobile phase was 0.1% formic acid aqueous solution (A) and acetonitrile (B). The gradient elution was: 0.01–5 min, 5%–10% B; 5–12 min, 10%–35% B; 12–20 min, 35%–80% B; 20–30 min, 80%–85% B; 30–35 min, 85%–95% B; 35–37.1 min, 95%–5% B. The flow rate was 0.25 ml/min, and 2 μL of filtrate was injected into the UPLC-QTOF-MS/MS system for analyses.

The instrument settings for the QTOF-MS/MS system were chosen carefully. The electrospray ionization (ESI) source was positive and negative modes. The positive and negative ionization spray voltages were 5,500 and 4,500V, respectively. The ion source temperature was 500°C. The declustering potential was 100 V. The collision energy was 40 eV. Ion source gas 1 (GS1) and GS2 were both set to 50 psi. The curtain gas was set to 40psi. Samples were analyzed in positive and negative ionization modes with a scanning mass-to-charge (*m/z*) range from 50 to 1,000. The collision energy spread was 15 V. Data were collected in information-dependent acquisition mode and analyzed by PeakView^®^1.2 (AB Sciex).

### ARC Components and Prediction of Candidate Compounds

An in-house database of the compounds contained in ARC was established. The information in this database was the name, molecular formula, molecular weight, characteristic fragment ions, and relevant references. An empirical molecular formula matching the criterion of <5 ppm was deduced as a potential candidate by PeakView (Ma et al., 2020). Only those compounds that had been compared with standards or characteristic fragment ions were finally selected as chemical composition of ARC. Then, we entered the Simplified Molecular Input Line Entry System (SMILES) format of the components present in ARC obtained from the PubChem database (https://pubchem.ncbi.nlm.nih.gov/) into the Molinspiration website (www.molinspiration.com/cgi-bin/properties) to calculate the prediction parameters for drug absorption (Ma et al., 2016). Next, we used the Lipinski Rule of five (Lipinski et al., 2001). That is, if a ARC component was subject to the following provisions of the corresponding parameters, it could be identified as an absorbable component: hydrogen-bond donor (number of hydrogen atoms attached to O and N) ≤5; relative molecular mass ≤500; partition coefficient of fat:water miLogP ≤5; hydrogen-bond acceptor (number of O and N) ≤10.

### Identification of the Related Targets of ARC Components

Established biological/chemoinformatics methods can aid prediction of the targets of chemical constituents in TCM formulations (Chaudhari et al., 2017; Byrne and Schneider, 2019)^.^ We obtained data from Traditional Chinese Medicine Systems Pharmacology (TCMSP, http://lsp.nwu.edu.cn/tcmsp.php) and SwissTargetPrediction (http://www.swisstargetprediction.ch/) databases for target collection.

### Acquisition of Targets for GA

We used “gouty arthritis” as the keyword in the GeneCards database (www.genecards.org/) and the Comparative Toxicogenomics Database (CTD, http://ctdbase.org/) to identify disease targets associated with GA.

### Construction of a Compound–Target Network

First, we intersected the obtained drug targets with the targets associated with a disease, and obtained a Venn diagram of the intersected targets. Then, we built a network of complex information based on interactions between the components and target. Next, we used Cytoscape v3.7.1 (www.cytoscape.org/) to undertake visual analyses of the C–T network (Otasek et al., 2019).

### Construction of a Protein–Protein Interaction Network

We introduced drugs intersecting with disease targets into Search Tool for the Retrieval of Interacting Genes/Proteins (STRING) v11.0 (https://string-db.org/) (Hsia et al., 2015). We selected the "Multiple proteins" option to search for these targets while setting the organism to *Homo sapiens* to obtain a PPI network.

### Pathway-Enrichment Analyses Using Gene Ontology and Kyoto Encyclopedia of Genes and Genomes Databases

We used “clusterProfiler” within R (R Project for Statistical Computing, Vienna, Austria) (Yu et al., 2012) for functional classification and identification of enriched gene clusters.

### Expression of Targets in Organs

TCM theory holds that the human body is a whole, and that the way to treat diseases is to regulate multiple organs to achieve a “peaceful” state (Yuan et al., 2020). We wished to clarify the relationship between ARC efficacy and organs. Hence, we imported the targets in the top-20 nodes of the PPI network into the BioGPS database (http://biogps.org/). The species we selected was “human”. We obtained the protein-expression datasets of related targets in different organs, and exported them to Heml 1.0 to generate organ-expression heatmaps of targets.

### Computational Validation of C–T Interactions

We used computer simulations to investigate the mode of action of an active compound in the drug and its target. We selected two specific compounds and two specific targets for study using molecular docking. Autodock 2.4.6 is a genetic algorithm-based program used for docking protein–ligand complexes. We obtained data from the Research Collaboratory for Structural Bioinformatics Protein Data Bank (PDB) (www.rcsb.org/). The X-ray crystal structures of MAPK14 and MMP9 were obtained. The PDB entry code of these two proteins was 5ET1 and 1L6J, respectively. For molecular docking, we employed a semi-empirical free-energy calculation to evaluate the energy matching between the compound and target. The more negative the docking score, the more favorable was the interaction of the complex (Namasivayam and Günther, 2007).

### Experimental Validation

#### *In Vitro* Experiment

##### Cell Culture

THP-1 cells were cultured in RPMI-1640 medium with 10% fetal bovine serum. Cells were maintained in an incubator at 37 C in an atmosphere of 5% CO_2_ and saturated humidity. The culture medium was replaced with complete culture medium every 2–3 days.

##### CCK-8 Assay for Cell Viability

THP-1 cells were harvested during the logarithmic growth phase and seeded into 96-well plates at 1 × 10^6^ cells/well. Cells were treated with PMA (100 nM) for 24 h to induce their differentiation into resting M0 macrophages. Fresh complete culture medium was added and cultured allowed for 24 h after washing cells with phosphate-buffered saline. Then, differentiated THP-1 cells were stimulated with MSU (25, 50, 100, 150, 200, 300, 400 or 500 mg/ml) and treated with aurantiamide acetate (AA) at a final concentration of 0, 20, 60, 80 or 100 µM as well as colchicine (positive control, 5 µM) simultaneously for 24 h at 37 C (Xiao et al., 2019). After treatment, 10 μL of CCK-8 was added to each well, and the plates were incubated for an additional 1.5 h. Absorbance at 450 nm was measured (using 600 nm as a reference wavelength) on a SpectraMax™ M2 reader (Molecular Devices, Silicon Valley, CA, United States). The culture medium without cells was used as a blank. Cell survival was calculated as: absorbance/absorbance of control × 100%.

##### Western Blotting

Total protein was extracted by incubation of cell pellets with RIPA lysis buffer and a protease inhibitor (phenylmethylsulfonyl fluoride). The protein concentration was determined using the BCA Protein Quantification Kit according to manufacturer instructions. Cell lysates containing equal amounts of protein were fractionated by sodium dodecyl sulfate-polyacrylamide gel electrophoresis on 10% gels and then transferred to polyvinylidene fluoride (PVDF) membranes. After blockade with 5% nonfat milk in Tris-buffered saline containing 0.1% Tween 20 for 0.5 h at room temperature, the PVDF membranes were incubated with primary antibodies overnight at 4 C and horseradish peroxidase-conjugated secondary antibody for 1 h at room temperature, respectively. Protein bands were probed with ECL Plus chemiluminescent reagent and exposed to the ChemiScope Mini 3,300 chemiluminescent imaging system (Qinxiang, Shanghai, China).

##### Real-Time Reverse Transcription-Quantitative Polymerase Chain Reaction

Total RNA was extracted with TRIzol^®^ Reagent (Thermo Scientific, Waltham, MA, United States), and reverse-transcribed with oligo-DT using HiScript™ Reverse Transcriptase (Vazyme, Beijing, China) according to manufacturer instructions. The primers used were synthesized by Beijing Ruixingke Biotechnology (Beijing, China). The sequences were (forward and reverse, respectively): 5”-CAT​CCC​ACA​ATG​GGC​TCT​GT-3” and 5”-CTC​TTC​ACT​TCC​TGC​CCA​CA-3” for Caspase-1; 5”-CCT​GGC​GCA​GGT​GGA​C-3” and 5”-GTA​GTG​GTG​TGC​CTT​CCT​CC-3” for SYK; 5”-GAT​CCC​CAG​GGC​TCA​AAC​AT-3” and 5”-GAA​AAG​GCG​CAG​TTT​ACG​CT-3” for PTGS2; 5”-AAT​GGG​CAG​CCG​TTA​GGA​AA-3” and 5”-GCG​CCC​AAT​ACG​ACC​AAA​TC-3” for the internal control (GAPDH).

The PCR conditions were: 95°C for 3 min, 94 C for 30 s, 60°C for 30 s, 72°C for 30 s, 35 cycles, and extension at 72°C for 10 min. Then, 6 μL of the PCR-amplification products were electrophoresed in 1.5% agarose gel, imaged and photographed under a gel imager (Liuyi, Beijing, China). GAPDH was used as an internal reference to determine relative mRNA expression of the genes to be measured. The 2^−△△CT^ method was the basis for measuring relative expression of genes.

#### *In vivo* Experiment

Thirty-two specific pathogen-free male Sprague−Dawley rats (180–220 g) were purchased from Nanjing Junke Bioengineering (Nanjing, China). After 1 week of adaptive feeding, 32 rats were divided randomly into four groups of eight: control; GA model (MSU); positive control (colchicine); ARC. The positive-control group was given colchicine (0.8 mg/kg i.g.). The ARC group was given an aqueous extract of ARC (0.3 g/kg i.g.). The control and MSU groups were given an equal volume of physiologic (0.9%) saline (i.g.) once a day for 7 days. On day-5, 200 μL of MSU (25 mg/ml) was injected into the knees of rats in the MSU group, colchicine group, and ARC group (Wang et al., 2019). In the control group, an equal volume of 0.9% saline was injected into rat knees.

Next, the circumference of the knee joint in rats was measured by using the tie line method for 0, 4, 6, and 24 h after MSU injection, respectively. The change in knee joint edema was calculated as follows: joint swelling = b–a. Here, a, b are the initial circumference of rat knee joint and the circumference after the injection of MSU crystal at each time-point, respectively.

On day-7, all rats in each group were anesthetized using 3% sodium pentobarbital. Blood was drawn from the abdominal aorta into a vacuum collection tube. Serum was obtained by centrifugation at 3,500 rpm/min for 10 min at 4°C. Levels of TNF-α, IL-1β, and IL-18 were measured by enzyme-linked immunosorbent assay (ELISA) kits. Synovium tissue samples were obtained from the joints of rats for histological analysis.

### Statistical Analyses

Data are the mean ± SD. The significance of results was determined based on one-way analysis of variance using Prism 8.0.1 (GraphPad, San Diego, CA, United States). *p* < 0.05 was considered significant. All experiments were repeated at least thrice.

## Results

### Identification of ARC Constituents by UPLC-QTOF-MS/MS

**FIGURE 2:**
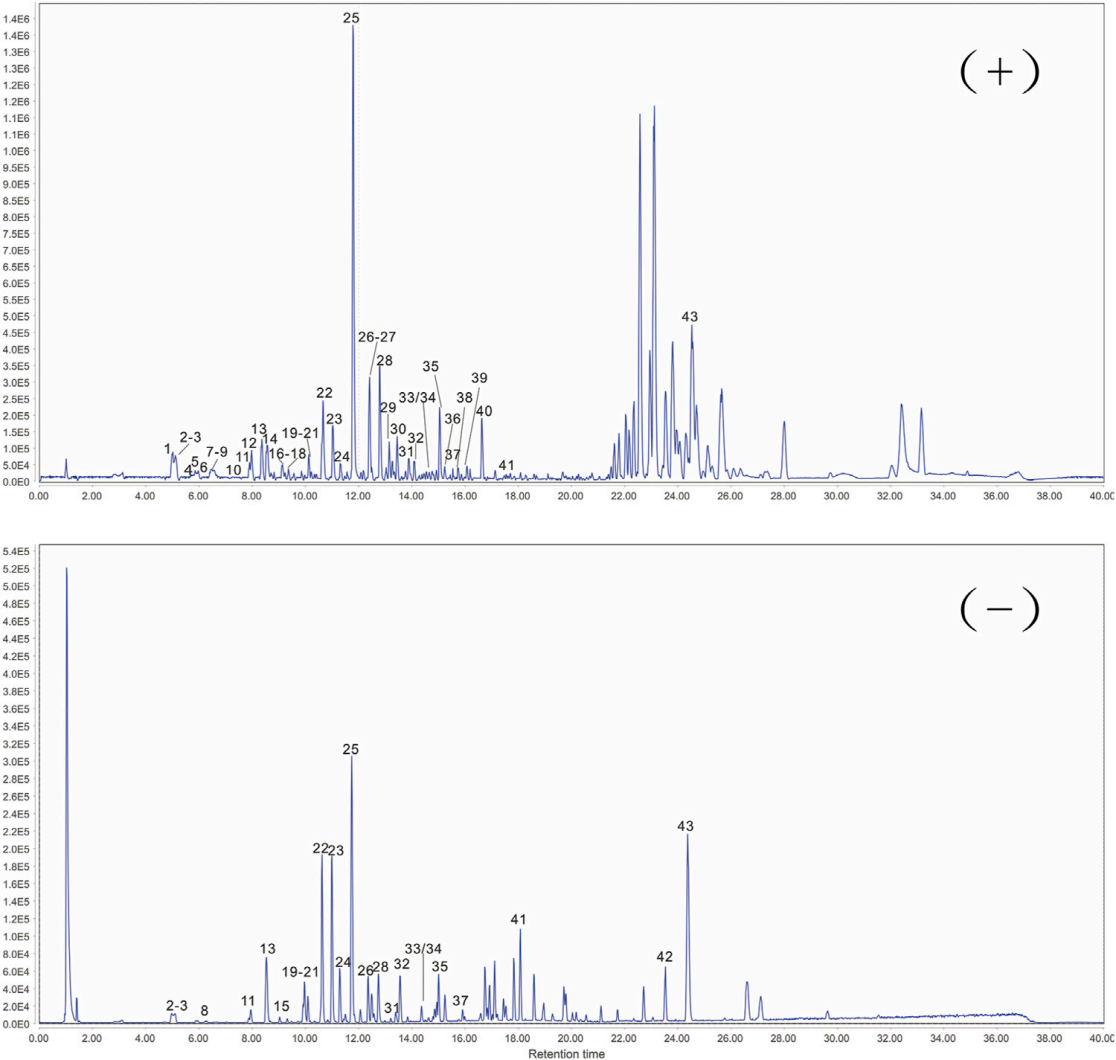
Representative base peak chromatogram of ARC in positive and negative ionization mode, respectively.

The response values of different chemical compositions differ in different modes. Hence, we undertook UPLC-QTOF-MS/MS in positive and negative ionization modes. Forty-three compounds were identified (Figure 2) and their results are shown in Table 1.

**TABLE 1:** Characterization of the chemical constituents in ARC by ultraperformance liquid chromatography quadrupole time-of-flight tandem mass spectrometry.

Number	t_R_/min	Formula	ESI-MS	ESI-MS/MS	ppm	Identity	References
1	4.88	C_22_H_33_NO_5_	[M + H] + 392.2424	374.2335, 356.2339	−1.8	Hokbusine B	Konno et al. (2004)
2	5.08	C_24_H_39_NO_9_	[M + H] + 486.2693	454.2433, 436.2327	−0.9	Mesaconine	Sun et al. (2013)
3	5.14	C_16_H_17_NO_3_	[M-H] − 270.1139	162.0584, 135.0498	1.5	Higenamine	Kosuge and Yokota (1976)
4	5.54	C_23_H_37_NO_6_	[M + H]+424.2688	406.2587	−1.2	Senbusine A	Sun et al. (2013)
5	5.77	C_22_H_35_NO_4_	[M + H] + 378.2633	360.2542, 342.2430	−1.3	Carmichaeline	Sun et al. (2013)
6	5.94	C_23_H_37_NO_5_	[M + H] + 408.2738	390.2641	−1.5	Isotalatizidine	Sun et al. (2013)
7	6.52	C_22_H_31_NO_3_	[M + H] + 358.2372	340.2268	−1.2	Songorine	Sun et al. (2013)
8	6.64	C_25_H_41_NO_9_	[M + H] + 500.2848	450.2492	−1.3	Aconine	Yang et al. (2014)
9	6.75	C_22_H_33_NO_3_	[M + H] + 360.2528	342.2434	−1.3	Napelline	Yang et al. (2014)
10	7.06	C_20_H_27_NO_3_	[M + H]+ 330.2059	330.2066	−1.3	Hetisine	Sun et al. (2013)
11	7.91	C_24_H_39_NO_8_	[M + H] + 470.2742	438.2489, 406.2230	−1.2	Hypaconine	Yang et al. (2014)
12	7.98	C_24_H_39_NO_7_	[M + H] + 454.2794	436.2691	−1.1	Fuziline	Sun et al. (2013)
13	8.38	C_24_H_39_NO_6_	[M + H] + 438.2845	420.2745, 388.2487	−1	Neoline	Sun et al. (2013)
14	8.52	C_25_H_41_NO_7_	[M + H] + 468.2947	418.2598	−1.7	Lycoctonine	Yuxin et al. (2016)
15	8.96	C_27_H_41_NO_8_	[M − H] − 506.2760	506.2703	0.2	Deltaline	Gardner et al. (1999)
16	9.01	C_23_H_37_NO_4_	[M + H]+ 392.2789	360.2520, 342.2434	−1.4	Sachaconitine	Yuxin et al. (2016)
17	9.13	C_24_H_39_NO_5_	[M + H] + 422.2894	358.2376	−1.5	Talatisamine	Sun et al. (2013)
18	9.16	C_22_H_33_NO_2_	[M + H] + 344.2578	326.2476	−1.7	Denudatine	Sun et al. (2013)
19	9.61	C_25_H_39_NO_7_	[M + H] + 466.2791	434.2536	−1.7	Delbruine	Yuxin et al. (2016)
20	9.73	C_26_H_41_NO_7_	[M + H] + 480.2948	462.2826	−1.6	14-Acetylneoline	Yuxin et al. (2016)
21	9.87	C_25_H_41_NO_6_	[M + H] + 452.2998	420.2750, 388.2421	−1.8	Chasmanine	Sun et al. (2013)
22	10.19	C_22_H_29_NO_3_	[M + H] + 356.2215	356.2224, 296.1606, 139.0203	−1.3	Songoramine	Yuxin et al. (2016)
23	10.34	C_29_H_39_NO_6_	[M + H] + 498.2844	480.2753, 462.2637	−1.1	Delavaconitine	Csupor et al. (2008)
24	11.06	C_27_H_43_NO_7_	[M + H] + 494.3104	462.2847	−1.6	Delbrusine	Yuxin et al. (2016)
25^a^	11.78	C_31_H_43_NO_10_	[M + H] + 590.2591	540.2561, 105.0345	−1.4	Benzoylmesaconine	Sun et al. (2013)
26^a^	12.41	C_32_H_45_NO_10_	[M + H] + 604.3104	572.2846, 554.2734	−1.9	Benzoylaconine	Yang et al. (2014)
27	12.6	C_27_H_31_NO_5_	[M + H] + 450.2264	450.2283	−2.3	Ignavine	Ochiai and Okamoto (2008)
28^a^	12.81	C_31_H_43_NO_9_	[M + H] + 574.3002	542.2738, 510.2468	−1.5	Benzoylhypacoitine	Yang et al. (2014)
29	13.35	C_33_H_45_NO_12_	[M + H] + 648.2998	588.2798	−2.4	Beiwutine	Yang et al. (2014)
30	13.72	C_33_H_45_NO_11_	[M + H] + 632.3055	572.2863, 540.2539	−1.6	Mesaconitine	Sun et al. (2013)
31	13.91	C_33_H_45_NO_9_	[M + H] + 600.3156	568.2886, 131.0491	−1.8	Isodelphinine	Katsui (2006)
32	14.21	C_34_H_47_NO_12_	[M + H] + 662.3163	602.2986	−1.1	10-OH-aconitine	Yuxin et al. (2016)
33	14.83	C_34_H_47_NO_11_	[M + H] + 646.3216	586.3030, 368.1870	−0.9	Aconitine	Sun et al. (2013)
34	14.83	C_33_H_45_NO_10_	[M + H] + 616.3101	556.2906, 524.2650, 338.1758	−2.4	Hypaconitine	Sun et al. (2013)
35	14.94	C_32_H_45_NO_8_	[M + H] + 572.3207	572.3221, 484.2699, 382.2020	−1.8	14-O-Anisoylneoline	Yuxin et al. (2016)
36	15.26	C_34_H_47_NO_9_	[M + H] + 392.2424	596.3221, 508.2669, 570.2681, 538.2787	−1.8	Chasmaconitine	Yang et al. (2014)
37	15.51	C_34_H_47_NO_10_	[M + H] + 630.3259	510.2822, 352.1917	−2.2	Deoxyaconitine	Sun et al. (2013)
38	15.85	C_41_H_43_NO_12_	[M + H] + 742.2845	620.2643	−1.7	Delgrandine	Yuxin et al. (2016)
39	16.21	C_35_H_49_NO_9_	[M + H] + 628.3466	596.3146	−2.2	Vilmorrianine C	Yuxin et al. (2016)
40	16.95	C_29_H_37_NO_5_	[M + H] + 480.2733	448.2468, 420.2493, 145.1025	−2.4	Cytochalasin B	Evidente et al. (2003)
41^a^	17.56	C_27_H_28_N_2_O_4_	[M − H] − 443.1981	443.0180, 59.0223	1.1	Aurantiamide acetate	Songue et al. (2012)
42	23.28	C_14_H_28_O_2_	[M − H] − 227.2023	227.2020, 183.0452	3.2	Myristic acid	Bianco et al. (2020)
43	24.38	C_18_H_32_O_2_	[M-H] − 279.2342	279.2339	4.5	Linoleic acid	Bianco et al. (2020)

a Identified by comparison with standards.

### Screening of the Active Constituents in ARC

The absorption parameters of the compounds identified in ARC were calculated by computer-based prediction methods. Twenty-five chemical constituents met the five principles of drug absorption (Table 2). Some constituents did not meet the screening criteria for constituents but may also have therapeutic effects on the human body. To investigate this issue more comprehensively, although they did not meet the screening criteria, these candidates were retained as active ingredients in our study. For example, benzoylmesaconine, benzoylaconine, and aconitine did not meet the screening criteria, but they were retained as active ingredients because they are the main components of ARC (He et al., 2017). Yu and colleagues suggested that benzoylaconine suppresses IL-1β-induced expression of IL-6 and IL-8 *via* inhibition of activation of mitogen-activated protein kinase (MAPK) (i.e., extracellular signal-regulated kinase (ERK), c-Jun N-terminal kinase (JNK), p38), protein kinase B (Akt) and nuclear factor-kappa B (NF-κB) pathways in SW982 human synovial sarcoma cells (Yu et al., 2020). Benzoylmesaconine exhibits analgesic, antiviral and antifungal activities (Dai et al., 2014). Aconitine has immunomodulatory properties and may be a potentially efficacious drug for treatment of autoimmune diseases (Li et al., 2017). Although several constituents had relatively low kinetic values, they are biologically active and could be considered to be candidates. We hypothesized that, as long as the candidate components in ARC intersected with the target of GA, they could be considered to be active components. In summary, 31 compounds were selected as active constituents in ARC (Supplementary Table S1).

**TABLE 2:** Absorption parameters of ARC components.

No.	Component	MW	miLogP	nON	nOHNH
1	Songorine	357.49	1.93	4	2
2	Talatisamine	421.58	1.93	6	2
3	Carmichaeline	377.52	1.87	5	3
4	Higenamine	271.32	2	4	4
5	Aurantiamide acetate	444.53	3.89	6	2
6	Cytochalasin B	479.62	4.11	6	3
7	Fuziline	453.58	0.1	8	4
8	Aconine	499.6	-1.15	10	5
9	Mesaconine	485.57	-1.52	10	5
10	Hypaconine	469.57	-0.61	9	4
11	Sachaconitine	391.55	2.49	5	2
12	Delbrusine	493.64	2.38	8	0
13	Neoline	437.58	1.01	7	3
14	Lycoctonine	467.6	0.68	8	3
15	Delbruine	465.59	1.15	8	2
16	Chasmanine	451.6	1.63	7	2
17	Songoramine	355.48	2.38	4	1
18	Denudatine	343.51	3.26	3	2
19	Senbusine A	423.55	0.4	7	4
20	Hetisine	329.44	1.31	4	3
21	14-Acetylneoline	479.61	1.72	8	2
22	Ignavine	449.55	2.8	6	3
23	Delavaconitine	497.63	3.7	7	2
24	Napelline	359.51	2.11	4	3
25	Hokbusine B	391.51	1.95	6	3

### Identification of the Related Targets of ARC Constituents

All the targets of the compounds in ARC were collected from SwissTargetPredition and TCMSP. After removing the redundant information, 31 components in ARC and 394 known targets associated with them were obtained (Supplementary Table S2).

### Acquisition of Known Therapeutic Targets for GA

After collecting and removing redundant items from GeneCards and CTD, 2000 known GA treatment targets were collected (Supplementary Table S3).

### Analyses of the C–T Network

**FIGURE 3:**
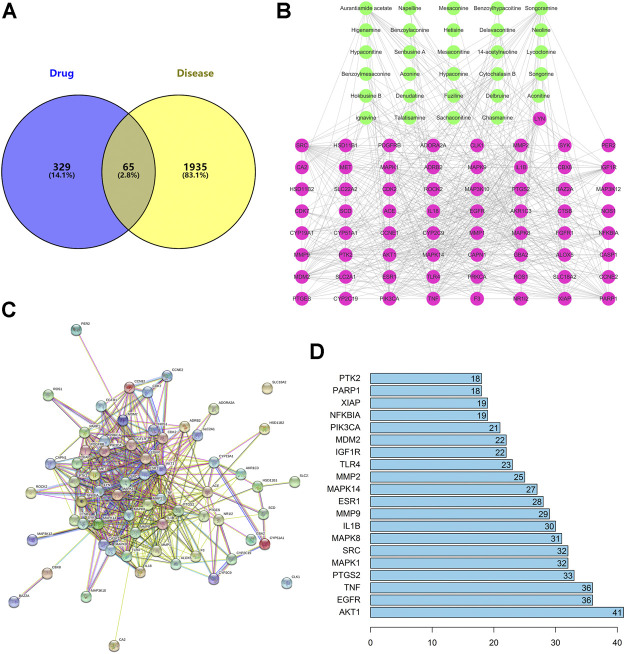
**(A)** There are 65 overlapping targets between disease and drug. **(B)** C–T network. The 29 green nodes indicate the active ingredients of GA for drug treatment. The 65 pink nodes indicate coincident targets between disease and drug. The edges denote that nodes can interact with each other. **(C)** PPI network. **(D)** Bar plot of the PPI network. The x-axis represents the number of neighboring proteins of the target protein. The y-axis represents the target protein.

There were 65 overlaps of 2000 targets for disease and 394 targets for the drug (Figure 3A). That is, 65 targets may be key for ARC in GA treatment. The 65 overlapping targets are detailed in Supplementary Table S4.

To elucidate how ARC acts on GA, we constructed a C–T network (Figure 3B). We identified 29 active components that had therapeutic effects on GA. The 29 green nodes represented active components in ARC; the 65 pink nodes represented overlapping targets between the disease and drug. Edges indicated that nodes could influence each other.

We undertook further network analyses by evaluating centralization and heterogeneity, which were 0.348 and 0.882, respectively. Therefore, some nodes were more concentrated in the network than others. These nodes may be more important and merited in-depth study. In addition, the network also included some components that had multiple targets: AA (degree = 18), songoramine (degree = 18), cytochalasin B (degree = 16), and ignavine (degree = 17). Hence, the same active component in ARC could act on multiple targets. Simultaneously, the same target could be affected by multiple components, such as AKT1 (degree = 44), caspase 1 (degree = 18), IL-18 (degree = 17) and SYK (degree = 15). This finding also illustrated, from another perspective, that TCM theory involves is a mode of action with multiple components and multiple targets. Details on the active ingredients and targets are provided in Supplementary Table S5.

### Analyses of the PPI Network

We constructed a PPI network consisting of 65 nodes and 443 edges (Figure 3C). This PPI network was based on the premise that proteins have more interactions among themselves than would be expected for a random set of proteins of similar size, drawn from the genome. Such enrichment indicates that the proteins are at least partially biologically connected, as a group.

The light-blue edges denote known interactions from curated databases. The pink edges show that the known interactions were determined by experimental methods. The green edges demonstrate that the predicted interactions arose from a neighboring gene. The red edges show that the predicted interactions arose from gene fusions. The dark-blue edges denote that the predicted interactions arose from gene co-occurrence. The yellow edges demonstrate that the predicted interactions arose from text-mining. The black edges denote that the predicted interactions arose from co-expression. The lavender edges show that the predicted interactions arose from protein homology. The details of the PPI network are described in Supplementary Table S6.

We took the top-20 proteins in the PPI network. AKT1 may be associated with 40 other proteins (Figure 3D). TNF and epidermal growth factor receptor (EGFR) were related to the other 35 proteins. PTGS2 was associated with 32 other proteins. These results suggested that these four proteins would be the focus of our study on PPIs. CLK1 and SLC18A2 were not associated with other proteins in this PPI network, implying that they were less important.

### Pathway-Enrichment Analyses Using GO and KEGG Databases

**FIGURE 4:**
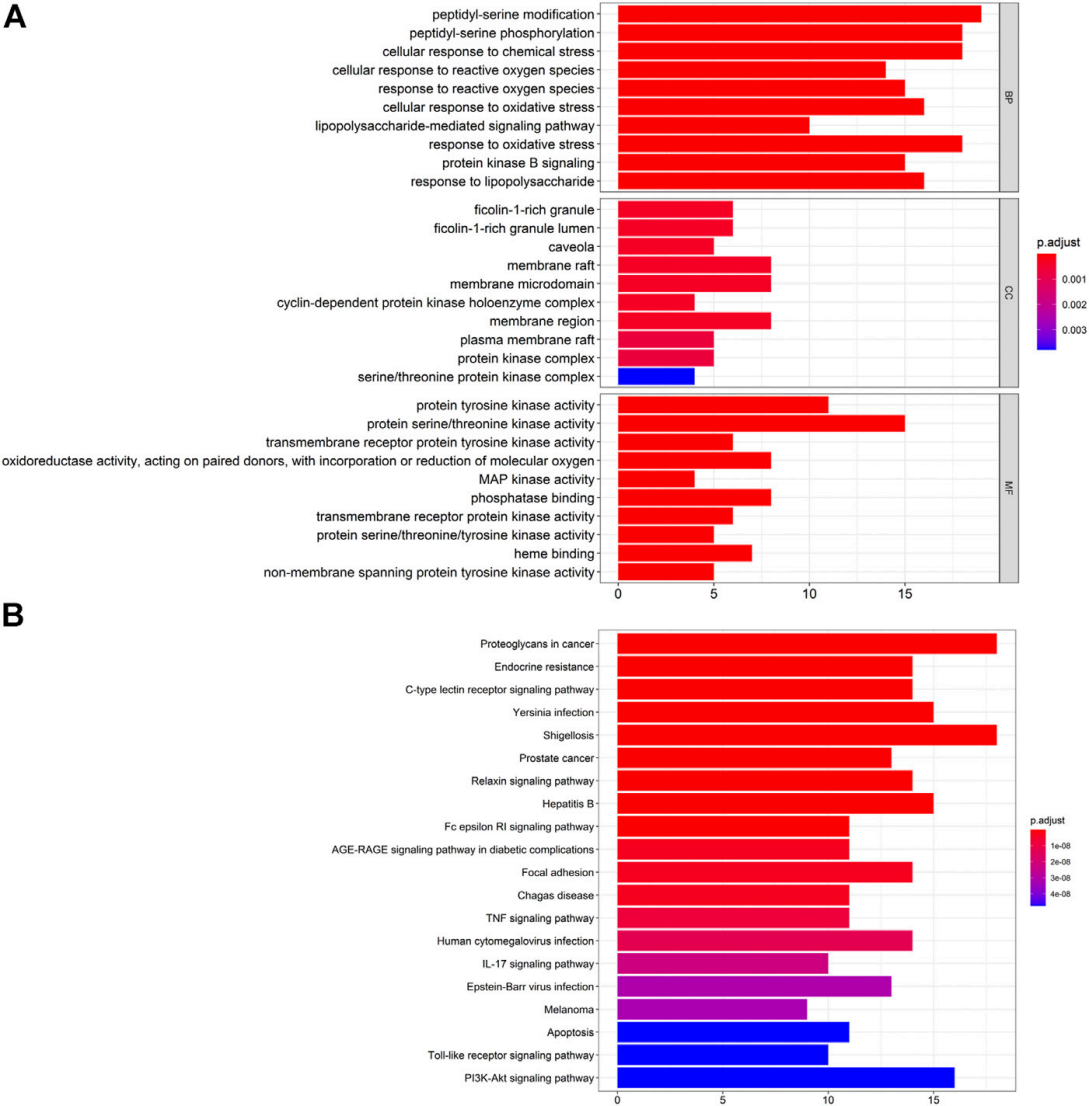
**(A)** Analyses of 65 targets associated with gouty arthritis using the GO database. The x-axis represents significant enrichment of these term counts. The y-axis represents the category of "biological process”, “cellular component” and “molecular function” in the GO of target genes (*p* < 0.05). **(B)** Pathway-enrichment analyses using the KEGG database. The x-axis represents the count of targets in each pathway; the y-axis represents the primary pathway (*p* < 0.01).

First, we undertook pathway-enrichment analyses using the GO database to elucidate the relevant biological processes (Figure 4A). The y-axis represents GO terms. The x-axis indicates the number of targets enriched in this term. The redder the color, the smaller is the value of p. adjust (false discovery rate (FDR)). It also denotes greater credibility and greater importance. In contrast, the bluer the color, the greater is the value of p. adjust. The top-ten terms of Biological Process (BP), Cellular Component (CC), and Molecular Function (MF) were screened for presentation for adjustment from small to large, respectively.

Details of pathway-enrichment analyses using the GO database are provided in Supplementary Table S7. The results showed that GA treatment involved multiple BPs, and BPs mainly involved peptidyl-serine modification (GO: 0018209), peptidyl-serine phosphorylation (GO: 0018105), and cellular response to chemical stress (GO: 0062197). CC mainly involved ficolin-1-rich granule (GO: 0101,002), ficolin-1-rich granule lumen (GO: 1904813) and caveola (GO: 0005901). MF mainly involved tyrosine kinase (GO: 0004713), protein activity/threonine kinase activity (GO: 0004674), and tyrosine receptor protein kinase (GO: 0004714).

Pathway enrichment analyses using the KEGG database are shown in Figure 4B. The y-axis represents the pathway. The x-axis indicates the number of targets enriched in this pathway. Sixty-five overlapping targets were mapped to 146 pathways after enrichment of pathways. For a simple demonstration, we can follow where the p. adjust intercepts the top-20 pathways from small to large. The details of KEGG pathway enrichment are shown in Supplementary Table S8.

Pathway enrichment analyses using the KEGG database indicated that ARC treatment of GA could affect the modules of “tumor”, “immune response”, and “inflammation”. Sixty-five overlapping targets were closely related to the relevant pathways of prostate cancer (hsa05215), C-type lectin receptor signaling pathway (hsa04625), TNF signaling pathway (hsa04668) and endocrine resistance (hsa01522) (Figure 4B). These pathways may be the key pathways responsible for GA treatment. This type of analysis provides a new way to elucidate the mechanism of action of ARC in GA treatment.

### Expression of Targets in Different Organs

**FIGURE 5:**
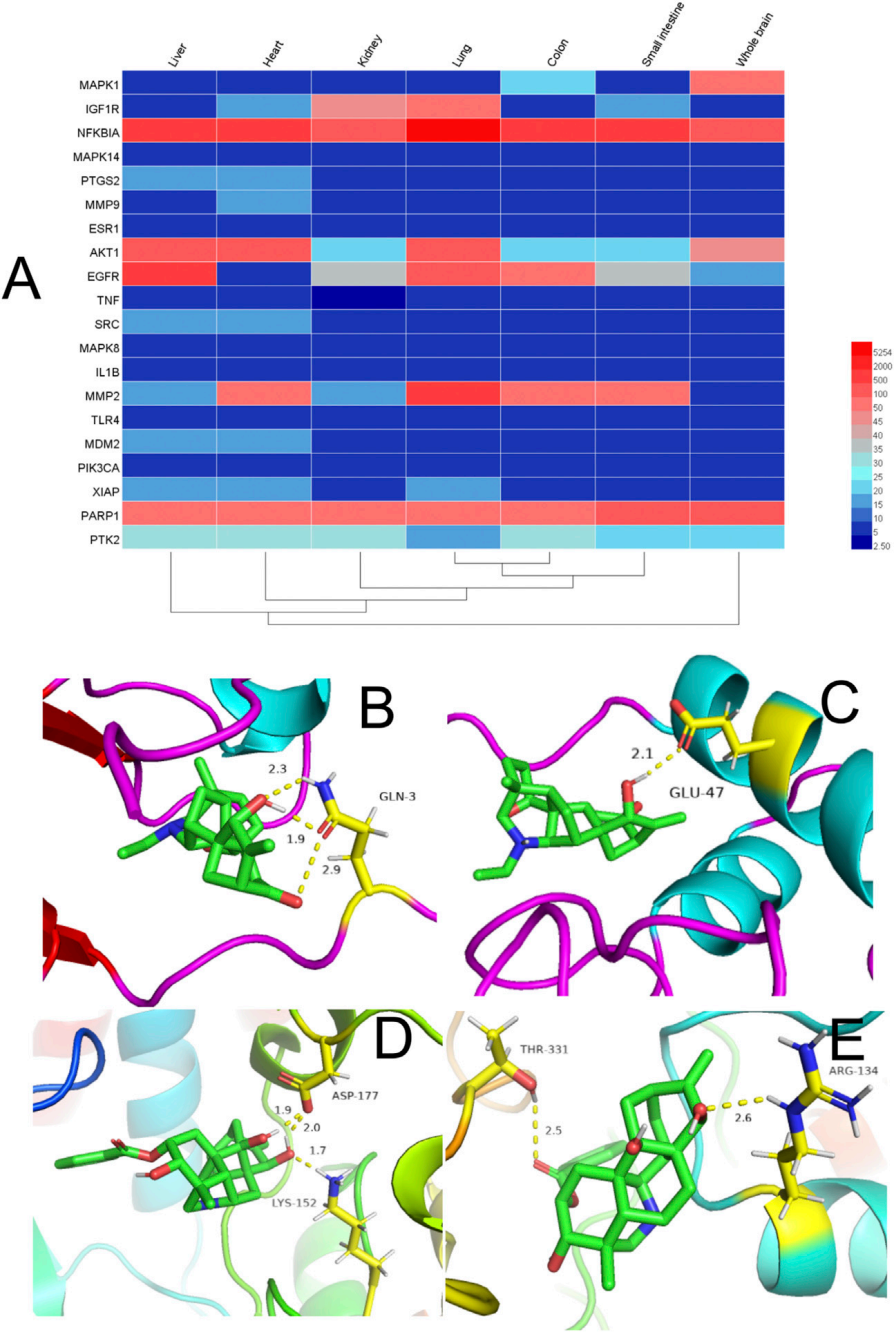
**(A)** Heatmap of organ expression of 20 targets. The x-axis indicates the organ name. The y-axis indicates the target name; from left to right, liver, heart, kidney, lung, small intestine, and whole brain. **(B-E)** Binding studies of selected compound–target interactions. **(B)** Songoramine with MAPK14. **(C)** Songoramine with MMP9. **(D)** Ignavine with MAPK14. **(E)** Ignavine with MMP9. Molecules are represented by a ball-and-stick model. Hydrogen bonds are represented by a dotted line, and numbers indicate hydrogen-bond distances in angstroms. Atoms C, O and N are green, red and blue, respectively.

Expression of the top-20 targets in the PPI-network node degree in different organs is shown in Figure 5A (Supplementary Table S9). The redder the color, the higher is the expression of the target. Conversely, the bluer the color, the lower is the expression of the target. Most of the targets had high expression in the liver, kidney, small intestine, lung, and heart. TCM theory suggests that the interconnection and mutual influence of different organs on physiology and disease should also be based on the whole when treating local lesions (Yuan et al., 2020).

The liver is the main site of catabolism of endogenous free purine bases. The small intestine is responsible for catabolism of purine bases from excessive dietary sources. The excretion of uric acid occurs primarily at three sites: kidney (urine), intestine (feces) and liver (bile). About 90% of uric acid is reabsorbed as it passes through renal glomeruli. When its production (and reabsorption) rate exceeds the excretion rate, accumulation occurs. In turn, this causes hyperuricemia and increases the number of uric-acid crystals, and leads ultimately to GA (Ristic et al., 2020).

We showed that therapeutic targets could exist in two or more organs simultaneously, and that multiple targets could be distributed in the same organ. This finding suggests that human organs are interconnected during the occurrence and progression of diseases, and that ARC may have a therapeutic role in GA through multiple targets and multiple pathways.

### Computational Assessment of Selected C–T Interactions

In general, the smaller the binding energy, the more stable the ligand binds to the receptor. Binding energy <0 indicates that the ligand molecule can bind spontaneously to the receptor molecule (Namasivayam and Günther, 2007). Therefore, we explored the interaction and binding modes between MMP9, MAPK14, and their active components by means of *in silico* simulations.

Previous analyses of C–T networks have revealed a close association of the component songoramine to GA targets. Therefore, we first undertook molecular docking of songoramine with MAPK14. The binding energy between songoramine and MAPK14 was −7.83 kcal/mol, which indicated the possibility of strong binding between them. Songoramine and MAPK14 formed a hydrogen bond at glutamate (GLU)-3 (Figure 5B). The molecular-docking results of songoramine with MMP9 showed that the binding energy between them was −6.28 kcal/mol, which suggested good binding activity. Figure 5C shows the formation of hydrogen bonds at glutamate (GLU)-47 between songoramine and MMP9.

As another active component of treatable GA in ARC, ignavine was docked with MAPK14 and MMP9, respectively. The binding energy between ignavine and MAPK14 and MMP9 was −5.71 and −5.81 kcal/mol, respectively. This result indicated a good possibility of binding between ignavine and two GA targets. Ignavine could form hydrogen bonds with MAPK14 and MMP9 at lysine (LYS)-152, aspartate (ASP)-177, threonine (THR)-331, and arginine (ARG)-134, respectively (Figures 5D,E).

Based on these data, we suggest that the interactions between these active ingredients and targets underlie their biological activities. It also means that ARC has multiple components and multiple targets.

### Experimental Evaluation

#### *In Vitro* Experiment

##### CCK-8 assa

**FIGURE 6:**
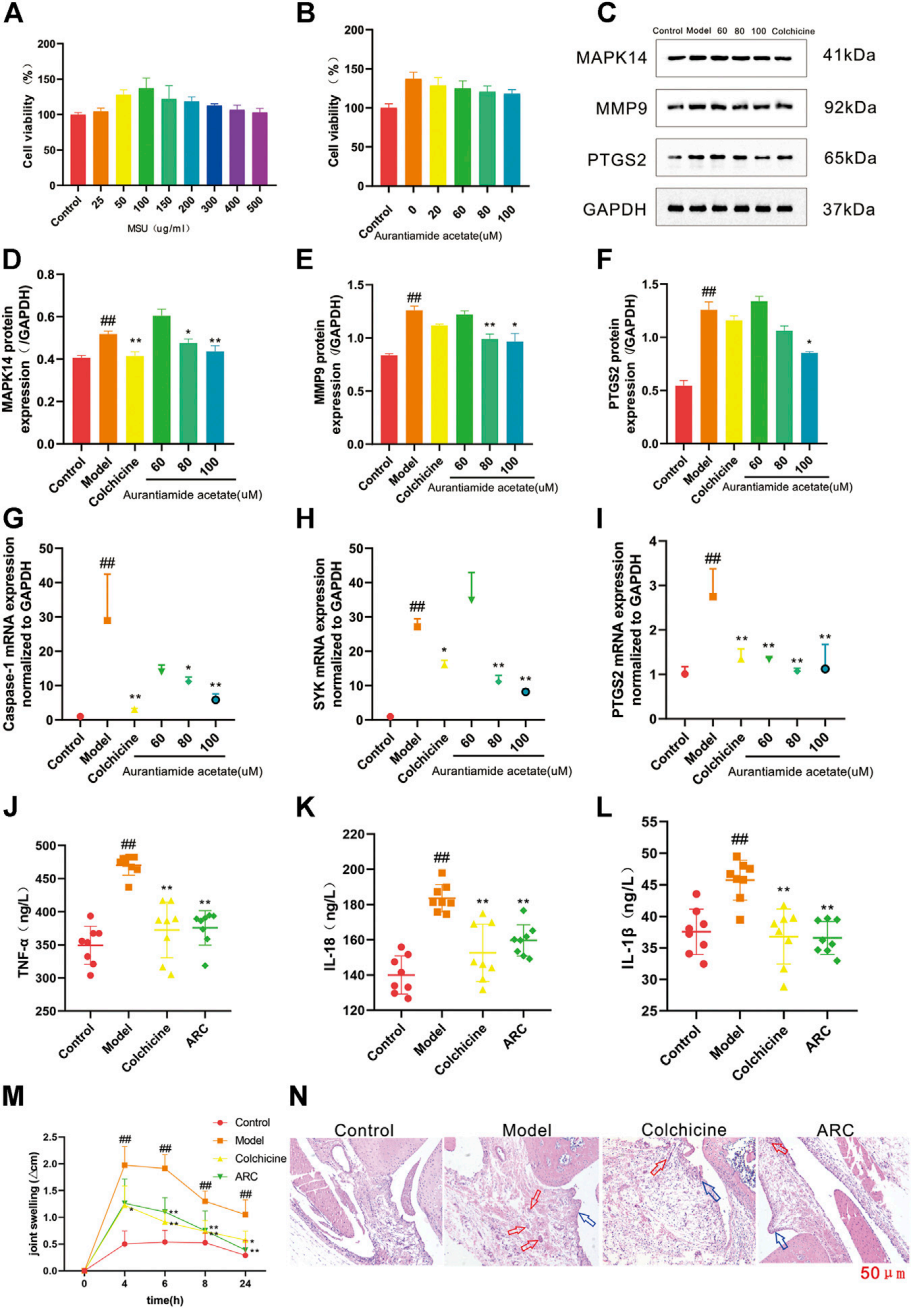
**(A)** THP-1 cells were exposed to MSU at various concentrations for 24 h. **(B)** Protective effects of AA on the viability of MSU-induced THP-1 cells. **(C)** Western blotting. **(D)** Expression of MAPK14 protein. **(E)** Expression of MMP9 protein. **(F)** Expression of PTGS2 protein. **(G)** Expression of caspase-1 mRNA. **(H)** Expression of SYK mRNA. **(I)** Expression of PTGS2 mRNA. **(J-L)** Effect of ARC on inflammatory factors in rat serum. **(J)** TNF-α. **(K)** IL-18. **(L)** IL-1β. **(M)** The knee joint circumference of rats were determined at 0, 4, 6, 8, 24 h after MSU stimulation, the increment at different time point was calculated. **(N)** Histopathological analysis of rat synovium tissue (100×). The red arrows pointed to inflammatory cells, blue arrows pointed to synovial hyperplasia. #*p* < 0.05, ##*p* < 0.01 vs. control group; *p < 0.05, **p < 0.01 vs. model group.

Cells were treated with MSU (25, 50, 100, 150, 200, 300, 400, or 500 μg/ml) to evaluate the activity of THP-1 cells (Figure 6A). Finally, 100 μg/ml of MSU was selected for induction in subsequent experiments. THP-1 cells treated with 20, 60, 80, or 100 μmol/L of AA showed viability in resistance to inflammatory activity of 118.2–128.7% (Figure 6B). To better demonstrate the effect of AA in GA treatment, we selected 60, 80, and 100 μmol/L for subsequent experiments.

##### Evaluation of Targets

We chose AA to test our predictions. Western blotting was done to measure expression of MAPK14, MMP9, and PTGS2. RT-qPCR was used to measure expression of caspase-1, SYK, and PTGS2 to confirm the previously predicted effect. Compared with that in the control group, expression of MAPK14, MMP9 and PTGS2 in THP-1 cells was increased significantly by MSU stimulation, and expression of MAPK14 and MMP9 was decreased significantly after treatment with AA (80 μmol/L); for PTGS2, it was decreased significantly after treatment with only a high dose of AA (Figures 6D–F).

RT-qPCR results showed that mRNA expression of caspase-1, SYK, and PTGS2 was increased significantly in the model compared with that in the control group, and expression of PTGS2 mRNA was decreased significantly in all dose groups. Expression of caspase-1 mRNA and SYK mRNA showed a significant decreasing trend upon treatment with AA at 80 μmol/L (Figures 6G–I).

The results of *in vitro* experiments showed that AA had a therapeutic effect on GA possibly by inhibiting inflammation thanks to regulation of the protein expression of MAPK14, MMP9, and PTGS2 and mRNA expression of caspase-1, SYK, and PTGS2.

### *In Vivo* Experiment

The MSU crystal-induced rats had a significant increase in knee joint circumference (Figure 6M). The intra-articular injection of MSU induced swelling of the knee joint and reached a maximum at 4–6 h, which then decreased significantly. The increase in knee joint circumference was significantly reduced in MSU crystal-induced rats when they were treated with ARC.

Furthermore, synovium tissue were stained with H&E and visualized via using a microscope (Figure 6N). In control group, synoviocytes were monolayer and abnormal inflammatory cells were not found in synovial tissue. The light-microscopic evaluation demonstrated a serious inflammatory reaction in the model group rats, within which synovial hyperplasia was obvious, cells arranged irregularly, massive inflammatory cell infiltrated, and cartilage erosion, while the damage of synovium tissue in ARC group were less severe, the synovial hyperplasia was inhibited and inflammatory response was diminished.

The proinflammatory cytokines IL-1β, IL-18, and TNF-α are the main cytokines involved in acute GA. Their expression can be used to characterize objectively inflammation severity in the body (Caution et al., 2019). To demonstrate the efficacy of ARC in GA treatment, we used ELISA kits to measure expression of proinflammatory factors in the serum of rats with MSU-induced GA. Compared with that in the control group, expression of IL-1β, IL-18, and TNF-α in the serum of rats was increased significantly by MSU stimulation, and expression of IL-1β, IL-18, and TNF-α was decreased significantly after treatment with ARC. ARC could reduce expression of these three proinflammatory factors in serum significantly, which confirmed our prediction results (Figures 6J–L).

## Discussion

The prevalence of GA seems to be increasing year-by-year worldwide (∼1–2% in 2018), especially in developing countries (Lyu et al., 2019). The etiology of GA is complex and the symptoms very diverse. ARC is highly toxic before processing and should not be eaten. The toxicity of ARC is reduced and its efficacy increased after processing. The ARC-induced toxicity is mostly caused by excessive or wrong medication (Wang et al., 2012). The primary target organ of raw root of *Aconitum carmichaeli* Debeaux toxicity is the heart. However, we have previously conducted cardiotoxicity studies of ARC. The ARC group was given an aqueous extract of ARC (0.3 g/kg i.g.). The control group were given an equal volume of physiologic (0.9%) saline (i.g.) once a day for 28 days. It was found that there was no statistically significant difference in the intensity of cardiotoxicity between ARC group and blank group, indicating that ARC was weakly or even non-toxic at reasonable doses (Ye et al., 2021). Colchicine is used commonly in GA treatment, but it has side effects. ARC is used often in TCM clinical practice to treat GA with considerable efficacy and fewer side effects, but some questions (e.g., relevant metabolites, underlying mechanism of action) remain unanswered (Nakao et al., 2011). Therefore, we investigated the mechanism of action of ARC in GA treatment by applying a systems-pharmacology approach. We combined the screening of active ingredients, drug targets, and methods of network/pathway analyses.

The aconitine found in ARC is used widely to treat pain and inflammation (Ameri, 1998). Ignavine can modulate opioid receptors by altering expression of the mRNA and protein of the factors involved in inflammation which, in turn, alleviates inflammation (Ohbuchi et al., 2016). AA can inhibit phosphorylation of p38 MAPKs to play a part in inflammation treatment (Yoon et al., 2014). AA can also inhibit PTGS2 expression (Martel-Pelletier et al., 2003; Zweers et al., 2011). PTGS2 plays an important part in inflammation. PTGS2 is expressed throughout inflammation in all systems, and produces different prostaglandins at different stages to have different roles (Kapoor et al., 2007; Mancini and Di Battista, 2011). We predicted that AA is an important active component of ARC in GA treatment and assessed the effectiveness of AA in GA treatment by *in vitro* cell experiments. Therefore, we suggest that one of the mechanisms of ARC in GA treatment may be through the inhibition of inflammation by AA.

Analyses of the PPI network revealed that MAPK1, PTGS2, MAPK14, IL-1β, MMP9, and TNF were the proteins with the top nodes. MAPK1 (also known as ERK2) is a proinflammatory cytokine that can lead to acute GA (Qian et al., 2020). MMP9 regulates inflammation after tissue injury by promoting immune-cell infiltration (Jing et al., 2020). PTGS2 is involved in immunomodulatory and inflammation-related signaling pathways, such as the C-type lectin receptor signaling pathway, TNF signaling pathway, and IL-17 signaling pathway (Kumar et al., 2014; Pinke et al., 2016). Proinflammatory cytokines such as IL-1β, IL-18, and TNF-α are the main cytokines involved in acute GA (Caution et al., 2019). In addition, there is a close link between MAPK14 targets and epithelial-cell signaling in *Helicobacter pylori* infection. Caspase-1 and SYK were relatively posterior in the PPI network, but several studies have shown that they are closely related to the occurrence and treatment of GA (Chung et al., 2019). Therefore, we believe that the targets stated above may be key to ARC in GA treatment. Molecular docking, *in vitro* cell experiments, and animal experiments demonstrated that ARC could inhibit inflammation by regulating the key targets stated above to treat GA.

*In vivo* experimental studies revealed that MSU crystal-induced knee joint swelling was significantly reduced in rats after treatment with ARC. At the same time, synovial tissue injury was mild, synovial hyperplasia was inhibited, and the inflammatory response was attenuated. IL-1β, IL-18, and TNF-α expression was significantly lower in serum.

The results of *in vitro* experiments showed that AA had a therapeutic effect on GA possibly by inhibiting inflammation thanks to regulation of the protein expression of MAPK14, MMP9, and PTGS2 and mRNA expression of caspase-1, SYK, and PTGS2.

By analyzing the enrichment of pathways using GO and KEGG databases, 1427 GO terms and 146 pathways were obtained. Among them, functional-enrichment results using the GO database were related mostly to peptidyl-serine modification, the cellular response to chemical stress, and protein-tyrosine-kinase activity in transmembrane receptors. Pathway-enrichment analyses showed that the active components of ARC could treat GA by regulating signaling pathways such as prostate cancer (hsa05215), C-type lectin receptor signaling pathway (hsa04625), TNF signaling pathway (hsa04668) and endocrine resistance (hsa01522). To a certain extent, these data also suggest that ARC has multiple components, multiple targets, and multiple pathways.

We propose that the following mechanisms may exist for ARC treatment of GA, and ARC can reduce joint swelling caused by MSU crystals, reduce synovial tissue damage, and decrease the expression of inflammatory factors in serum. AA in ARC may inhibit inflammation by regulating the protein expression of MAPK14, MMP9, and PTGS2 and the mRNA expression of caspase-1, SYK, and PTGS2.

In China, ARC is used commonly to treat rheumatic fever, joint pain, inflammation, and edema (Wen et al., 2016). However, its toxicity means that it cannot be eaten. Processing of ARC can reduce its toxicity and enhance its pharmacologic effects, so that it can be used orally by patients. We explored, systematically, how ARC may operate in terms of GA treatment. Our data may offer insights into how TCM can be employed in GA treatment.

## Data Availability

The original contributions presented in the study are included in the article/Supplementary Material, further inquiries can be directed to the corresponding authors.
